# Electrophysiological biomarkers and age characterize phenotypic heterogeneity among individuals with major depressive disorder

**DOI:** 10.3389/fnhum.2022.1055685

**Published:** 2023-01-09

**Authors:** Alexandra P. Key, Tricia A. Thornton-Wells, Daniel G. Smith

**Affiliations:** ^1^Department of Hearing and Speech Sciences, Vanderbilt University Medical Center, Nashville, TN, United States; ^2^Translational Medicine, Pharmaceutical and Early-Stage Clinical Development, Alkermes, Inc., Waltham, MA, United States

**Keywords:** biomarker, event-related potential, depression, age, N1, oddball, P300

## Abstract

**Introduction:** Despite the high need for effective treatments for major depressive disorder (MDD), the development of novel medicines is hampered by clinical, genetic and biological heterogeneity, unclear links between symptoms and neural dysfunction, and tenuous biomarkers for clinical trial contexts of use.

**Methods:** In this study, we examined the International Study to Predict Optimized Treatment in Depression (iSPOT-D) clinical trial database for new relationships between auditory event-related potential (ERP) responses, demographic features, and clinical symptoms and behavior, to inform strategies for biomarker-driven patient stratification that could be used to optimize future clinical trial design and drug development strategy in MDD.

**Results:** We replicate findings from previous analyses of the classic auditory oddball task in the iSPOT-D sample showing smaller than typical N1 and P300 response amplitudes and longer P300 latencies for target and standard stimuli in patients with MDD, suggesting altered bottom-up sensory and top-down attentional processes. We further demonstrate that age is an important contributor to clinical group differences, affecting both topographic distribution of the clinically informative ERP responses and the types of the stimuli sensitive to group differences. In addition, the observed brain-behavior associations indicate that levels of anxiety and stress are major contributing factors to atypical sensory and attentional processing among patients with MDD, particularly in the older subgroups.

**Discussion:** Our novel findings support the possibility of accelerated cognitive aging in patients with MDD and identify the frontal P300 latency as an additional candidate biomarker of MDD. These results from a large, well-phenotyped sample support the view that heterogeneity of the clinical population with MDD can be systematically characterized based on age and neural biomarkers of sensory and attentional processing, informing patient stratification strategies in the design of clinical trials.

## Introduction

Major depressive disorder (MDD) is a chronic and recurrent condition with a high and rising prevalence that is among the most burdensome global illnesses (Proudman et al., 2021). It negatively affects daily living, quality of life, cognition, employment, and productivity and is associated with myriad medical and mental health conditions. Antidepressant medications and cognitive-behavioral therapies are effective for some, but many patients (estimates range from 20% to more than 50%; Joffe et al., 1996) do not respond, are treatment-resistant, or have persistent deficits that detract from daily functioning (Kessler et al., 2003; Rush et al., 2006). A high degree of heterogeneity in clinical presentation, an expansive diversity of genetic, neuropathological, or neurophysiological underpinnings, and a lack of informative biomarkers hamper the certainty of diagnosis of MDD and its successful treatment (Fried, 2015). It is widely recognized that expanding the range of objective measures to improve MDD diagnosis, stratify patients, and predict treatment responsiveness or long-term outcomes may increase the probability of success in drug development and effective management of MDD (Buch and Liston, 2021).

Greater affordability and accessibility of functional neuroimaging techniques have led to a significant increase in the interest to identify objective brain-based measures that could predict risk, assist with a diagnosis, or evaluate treatment effects in clinical populations. Event-related potentials (ERP)—brief changes in ongoing EEG following a stimulus event—offer an effective means to noninvasively monitor brain functioning with millisecond-level precision. Differences in the amplitude, latency, and scalp distribution of various ERP responses are interpreted to index neural activity underlying sensory, perceptual, and higher-order cognitive processes and can be used to detect alterations associated with clinical diagnoses, including MDD (Mumtaz et al., 2015; Kangas et al., 2022).

To date, the auditory P300 ERP response has received the most attention as a possible biomarker of MDD-related alterations in neural function (e.g., Mumtaz et al., 2015). It is a positive peak with a centro-parietal scalp maximum occurring 300 ms after the stimulus onset and characterized by a larger amplitude in response to an infrequent target than the frequent standard stimulus in an oddball paradigm. The P300 is interpreted to reflect active attention allocation and working memory (Polich, 2012), both of which may be disrupted in MDD (e.g., Landrø et al., 2001; Hasler et al., 2004; Bruder et al., 2012; Rock et al., 2014). Several prior auditory P300 studies in MDD have reported reduced amplitudes to target tones (Gangadhar et al., 1993; Röschke and Wagner, 2003; Urretavizcaya et al., 2003; Kawasaki et al., 2004; Bruder et al., 2009; Kemp et al., 2009, 2010; Zhou et al., 2019) or a longer P300 peak latency (Bruder et al., 1991; Vandoolaeghe et al., 1998; Urretavizcaya et al., 2003; Houston et al., 2004). Yet, others noted no statistically significant differences in the P300 response characteristics between participants with MDD and healthy controls (Giedke et al., 1981; Sara et al., 1994; Ancy et al., 1996; Bruder et al., 1998; Kaustio et al., 2002; Nan et al., 2018). Studies examining the P300’s ability to predict treatment response also yielded inconsistent results: larger pre-treatment amplitudes or prolonged latencies were associated with better outcomes for antidepressant and electroconvulsive therapy treatments (Gangadhar et al., 1993; Bruder et al., 1995; Ancy et al., 1996; Vandoolaeghe et al., 1998) but did not predict treatment response to yoga (Murthy et al., 1998). Amplitude and latency of the P300 response were related to increased suicidal behavior (Hansenne et al., 1996), heightened anhedonia associated with the melancholic subtype (Schlegel et al., 1991; Ancy et al., 1996; Urretavizcaya et al., 2003), and the presence of psychotic features (Karaaslan et al., 2003) or cognitive impairment (Bruder et al., 1991; Vandoolaeghe et al., 1998). Direct associations between the P300 metrics and the severity of depression symptoms were detected in some (Nan et al., 2018) but not in other studies (Schlegel et al., 1991).

The auditory stimuli in the oddball paradigm elicit other ERP responses in addition to the P300, including the N1, a negative peak with a frontal or central scalp maximum occurring around 100 ms after sound onset and modulated by its physical characteristics. It reflects stimulus detection and early perceptual feature analysis (Näätänen and Picton, 1987). The N1 is generated in the primary auditory cortex (Pantev et al., 1990; Key et al., 2005), which is densely innervated by serotonergic neurons and characterized by a high serotonin synthesis rate (Brown et al., 1979; Gallinat et al., 2000). To date, examination of the N1 response using the auditory oddball paradigm in MDD yielded inconsistent findings. Several studies have reported that MDD patients elicit smaller or slower than typical N1 responses (Burkhart and Thomas, 1993; Urretavizcaya et al., 2003; Kemp et al., 2009, 2010), while others found no significant group differences (Sara et al., 1994; Greimel et al., 2015). Delayed latencies of the N1 or P300 also did not correlate with the behavioral ratings of depression symptoms (Urretavizcaya et al., 2003).

This variability in the N1 and P300 findings could be due to the small sample sizes and discrepant consideration of individual differences in the MDD group. In a large sample of MDD patients (*n* = 1,008), van Dinteren et al. (2015) observed smaller than typical P300 amplitudes to targets. Depression severity (Kemp et al., 2009; Jandl et al., 2010), years since diagnosis, and the duration of medication use (Houston et al., 2004), as well as age, have been identified as potentially important factors contributing to the conflicting ERP findings (Greimel et al., 2015). For example, only females with MDD younger than 46 years of age had slower P300 latencies compared to female healthy controls of the same age, while smaller than typical N1 amplitudes were observed only in young vs. older patients with MDD and in male non-responders vs. responders to venlafaxine XR (van Dinteren et al., 2015). Thus, it remains unclear whether the P300 or N1 responses reflect more general deficits associated with MDD (e.g., reduced motivation to engage with a task, Kähkönen et al., 2007) or are related to specific demographic or clinical features.

In the current study, we re-analyzed the large sample of well-characterized adults with MDD and age- and sex-matched healthy controls originally used by van Dinteren et al. (2015) to systematically examine whether the N1 and P300 responses to both target and standard stimuli in an auditory oddball task differentiate participants with MDD from healthy controls and whether they are sensitive to individual differences in age, biological sex, depression symptoms and severity, and factors related to daily functioning (anxiety, negativity bias, etc.).

## Materials and Methods

### Participants

The international multi-center, randomized, prospective open-label trial dataset (International Study to Predict Optimized Treatment Response in Depression, iSPOT-D; Williams et al., 2011) was previously described in van Dinteren et al. (2015) and included adults 18–65 years of age: 954 MDD patients and 328 healthy controls matched on age and sex. Data from the baseline (pre-treatment) study visits are included in the current analysis. Usable ERP data were available for 794 MDD patients and 307 healthy controls. A summary of the participant characteristics is presented in Table 1. The participants were not taking medication at the time of data collection.

**Table 1:** Demographic and clinical characteristics of the iSPOT-D study participants used in the current analysis.

		**Healthy controls**		**MDD**	
		**M**	**SD**	**M**	**SD**
**18–30 years**	N (F/M)	135 (79/56)		316 (188/128)	
	Age (years)	23.65	3.27	24.39	3.32
	Education (years)	14.93	2.45	14.83	2.61
	MDD duration (years)			7.83	5.16
	HDRS	1.14	1.48	21.65	4.01
	SOFAS	91.67	5.23	56.86	8.47
	DASS Anxiety	0.90	1.78	9.79	7.27
	DASS Depression	1.09	2.21	22.34	9.14
	DASS Stress	2.84	3.25	18.93	8.65
	BRISC Emotional Resilience	53.28	5.41	42.69	6.42
	BRISC Negativity Bias	92.46	4.99	62.11	12.67
	BRISC Social Skills	41.46	4.38	35.32	5.82
**31–45 years**	N (F/M)	77 (41/36)		226 (118/108)	
	Age (years)	38.43	4.27	38.27	4.16
	Education (years)	14.91	2.37	14.25	2.80
	MDD duration (years)			14.99	10.06
	HDRS	0.99	1.57	21.31	3.62
	SOFAS	88.10	12.50	56.74	9.91
	DASS Anxiety	0.48	0.93	7.27	5.67
	DASS Depression	0.93	1.57	21.87	9.37
	DASS Stress	3.03	3.43	16.99	7.79
	BRISC Emotional Resilience	53.07	5.02	43.74	6.18
	BRISC Negativity Bias	92.89	4.52	65.63	11.54
	BRISC Social Skills	40.61	3.89	33.41	6.47
**46–65 years**	N (F/M)	95 (54/41)		252 (135/117)	
	Age (years)	52.76	5.26	53.03	5.23
	Education (years)	15.04	2.67	14.37	2.96
	MDD duration (years)			22.77	15.63
	HDRS	1.24	1.69	22.02	4.06
	SOFAS	89.11	7.47	55.72	10.17
	DASS Anxiety	0.78	2.26	8.26	6.08
	DASS Depression	1.18	2.87	21.93	9.76
	DASS Stress	2.83	3.54	17.23	7.85
	BRISC Emotional Resilience	54.85	5.13	45.56	6.32
	BRISC Negativity Bias	93.36	5.60	65.87	12.24
	BRISC Social Skills	39.78	4.62	34.11	5.77
**Combined Sample**	N (F/M)	307 (174/133)		794 (441/353)	
	Age (years)	36.36	13.17	37.43	12.78
	Education (years)	14.96	2.49	14.52	2.79
	MDD duration (years)			14.58	12.48
	HDRS	1.13	1.57	21.67	3.92
	SOFAS	89.98	8.39	56.47	9.45
	DASS Anxiety	0.76	1.79	8.59	6.55
	DASS Depression	1.08	2.30	22.08	9.40
	DASS Stress	2.88	3.37	17.84	8.20
	BRISC Emotional Resilience	53.71	5.27	43.90	6.43
	BRISC Negativity Bias	92.84	5.08	64.30	12.34
	BRISC Social Skills	40.74	4.39	34.40	6.04

MDD, major depressive disorder; HDRS, Hamilton Depression Rating Scale; SOFAS, Social and Occupational Functioning Assessment Scale; DASS, Depression Anxiety and Stress Scales; BRISC, Brain Resource Inventory of Social Cognition.

### Clinical assessments

All participants completed the 17-item *Hamilton Rating Scale for Depression* (HRSD; Hamilton, 1986), a clinician-administered self-report assessment of symptoms of depression experienced over the past week. A score of 0–7 is considered to be within the normal range (or in clinical remission), while a score of 20 or higher indicates at least moderate severity of depressive symptoms.

The *Social and Occupational Functioning Assessment Scale* (SOFAS; American Psychiatric Association, 1994) is a clinician-completed global rating measure of current functioning ranging from 0 to 100, with lower scores representing lower functioning (Goldman et al., 1992).

A self-report on the *Quick Inventory of Depressive Symptomatology* (QIDS-SR16; Rush et al., 2003) documented depressive symptom severity with the total score ranging from 0 to 27, where scores of 5 or lower indicate no depression, scores from 6 to 10 correspond to mild depression, 11–15—moderate depression, 16–20—severe depression, and 21–270—very severe depression.

The *Depression Anxiety and Stress Scales* (DASS; Lovibond and Lovibond, 1995) included 42 self-report items measuring depression, anxiety, and stress, with higher scores indicating greater severity of symptoms.

Participants also completed the *Brain Resource Inventory of Social Cognition* (BRISC; Gordon et al., 2008) for assessment of self-regulation processes that included 45 self-report items related to Negativity Bias, Emotional Resilience, and Social Skills. Lower scores indicate less efficient functioning.

### ERP procedures

Detailed data acquisition and processing procedures are described in van Dinteren et al. (2015). Briefly, the auditory event-related potentials (ERPs) were recorded using an oddball paradigm with a 500 Hz tone as the standard condition (280 trials) and a 1,000 Hz tone as the target (60 trials). All tones were 50 ms long (5 ms rise/fall time), presented binaurally in random order at 75 dB sound pressure level, with 1,000 ms interstimulus interval. Two targets could not appear consecutively. Participants pressed two buttons simultaneously with the index fingers of each hand in response to the target tones. Speed and accuracy of response were both equally emphasized, and a brief practice session familiarized the participants with the stimuli and the task.

ERPs were acquired using 26 Ag/AgCl electrodes (Quikcap with the extended International 10–20 electrode placement system: Fp1, Fp2, F7, F3, Fz, F4, F8, FC3, FCz, FC4, T3, C3, Cz, C4, T4, CP3, CPz, CP4, T5, P3, Pz, P4, T6, O1, Oz, O2) and Neuroscan NuAmps DC amplifier with 500 Hz sampling rate. Additional electrodes placed at the outer canthus of each eye as well as above and below the left eye monitored horizontal and vertical eye movements with a ground at AFz. Impedances were kept at or below 5 kOhms. Data preprocessing was completed by the original iSPOT-D study team using BrainVision Analyzer 2 and custom automated pipelines validated against manual review (for specific details see van Dinteren et al., 2015; Arns et al., 2016). Briefly, data were re-referenced offline to averaged mastoids, low-pass filtered at 35 Hz with a Tukey (tapered cosine) window, and baseline corrected using the 300-ms prestimulus interval. Ocular artifacts were corrected using a regression-based technique similar to that by Gratton et al. (1983).

### Statistical analyses

Statistical analysis included only the correct response trials. In the original report aiming to identify neural predictors of pharmacological treatment outcomes in MDD (van Dinteren et al., 2015), the target stimulus waveforms at frontal (Fz) and parietal (Pz) locations were analyzed relative to a 300 ms pre-stimulus baseline. In the current analysis, peak amplitude and latency measures for the N1 (70–120 ms) and P300 (300–500 ms) were examined for both stimulus conditions (standard and target tones) for a more detailed evaluation of auditory processing and to assess stimulus discrimination effects in MDD. In line with the published guidelines for clinical research using ERPs (Duncan et al., 2009), amplitude and latency measures were obtained at frontal, central, and parietal midline electrodes (Fz, Cz, Pz) to provide a more detailed evaluation of the topographic distribution of the N1 (fronto-central maximum, Näätänen and Picton, 1987) and P300 (parietal maximum for the goal-directed attention, frontal maximum for the involuntary orienting; Polich, 2007) as well as to account for possible differences in neural processing between the clinical groups. The time window for the P300 response was adjusted from the wide interval (220–550 ms) in van Dinteren et al. (2015) to the more traditional 300–500 ms window to minimize the potential overlap with the preceding auditory P200 response, which may also be affected in MDD patients (e.g., Vandoolaeghe et al., 1998; Chen et al., 2002).

Examination of the age distribution in the study sample revealed a non-normal distribution, with greater representation of the younger age range (18–30 years), and a median of 46 years. Therefore, for the purpose of analyses, the sample was divided into three age subgroups: 18–30 years, 31–45 years, and 46–65 years. These subgroups represent commonly used divisions in the field as well as reflect known age-related differences. Previously, age 30 has been reported as the end point of neural development, followed by a plateau and then degeneration (van Dinteren et al., 2014), and aging-related alterations in neural activity can be seen after age 45 years (Handy, 2005).

The statistical analyses focused on Group differences (MDD vs. healthy controls) in the auditory ERP characteristics using repeated measures ANOVA with Stimulus (2: standard, target) × Electrode (3: Fz, Cz, Pz) within-subject factors and Group (2: Control, MDD) × Sex (2: female, male) × Age subgroup (3: 18–30, 31–45, 46–65 years) as the between-subject factors. Huynh-Feldt correction was used for sphericity violations. Only main effects and interactions involving Group or Stimulus factors were considered for interpretation. Brain-behavior associations were examined in the combined sample representing the full continuum of depressive symptoms using correlations between the ERP measures sensitive to group differences and the clinical data related to depression presence and severity.

## Results

**Figure 1:**
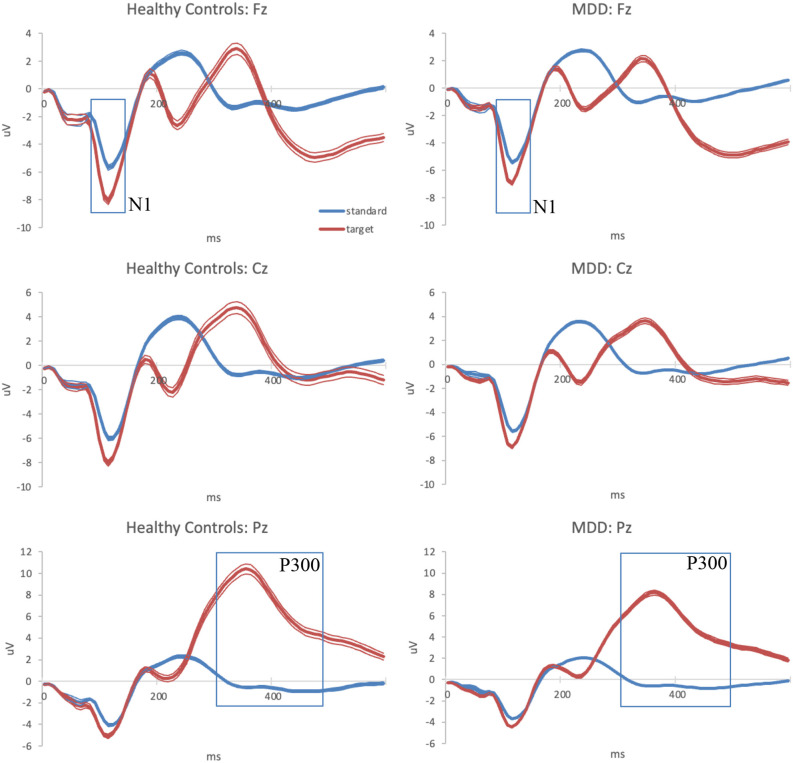
Grand-average auditory ERPs for standard (blue) and target (red) tones recorded during the baseline visit in healthy controls (left column) and MDD patients (right column) at frontal, central, and parietal midline scalp locations. Standard errors are plotted as thin lines around each waveform. MDD, major depressive disorder; ERPs, event-related potentials.

The averaged ERP waveform for each group at the three analyzed scalp locations is presented in Figure 1.

### N1 amplitude

There were main effects of Group, *F*_(1,1089)_ = 18.236, *p* < 0.001, $\eta_{p}^{2}$ = 0.016; Sex, *F*_(1,1089)_ = 8.04, *p* = 0.005, $\eta_{p}^{2}$ = 0.007; and Stimulus, *F*_(1,1089)_ = 284.458, *p* < 0.001, $\eta_{p}^{2}$ = 0.207. Significant interactions included Stimulus × Group, *F*_(1,1089)_ = 11.411, *p* < 0.001, $\eta_{p}^{2}$ = 0.001; Stimulus × Electrode, *F*_(2,2178)_ = 54.303, *p* < 0.001, $\eta_{p}^{2}$ = 0.047; and Stimulus × Electrode × Group × Age, *F*_(4,2178)_ = 3.742, *p* = 0.005, $\eta_{p}^{2}$ = 0.007.

Further analyses of the interactions indicated that both study groups differentiated between the standard and target stimuli, with larger amplitudes for the latter (*p* < 0.001; controls *d* = 0.780, MDD *d* = 0.448). However, the MDD group generated smaller (less negative) N1 responses than the healthy controls for both standard, *F*_(1,1203)_ = 4.698, *p* = 0.030, and target stimuli, *F*_(1,1116)_ = 368.581, *p* < 0.001. The overall magnitude of stimulus discrimination quantified as the amplitude difference between the target and standard stimuli was also significantly smaller in the MDD group than in the healthy controls, *F*_(1,1100)_ = 14.674, *p* < 0.001 (M = 1.2 μV vs. 1.9 μV).

Consideration of the age-related effects noted that within each age subgroup, all participants differentiated between the standard and target tones (*p* < 0.001). In the 18–30-year-olds, clinical group differences were observed in response to target but not to standard tones with larger amplitudes in the healthy controls than the MDD group at all three analyzed scalp locations: Fz: *F*_(1,461)_ = 7.950, *p* = 0.005; Cz: *F*_(1,486)_ = 17.132, *p* < 0.001; Pz: *F*_(1,492)_ = 12.257, *p* < 0.001. A similar pattern was observed in the 31–45-year-olds, Fz: *F*_(1,314)_ = 11.068, *p* < 0.001; Cz: *F*_(1,334)_ = 6.311, *p* = 0.012; Pz: *F*_(1,339)_ = 4.059, *p* = 0.021. In the oldest subgroup, 46–65 years, clinical group differences included the N1 amplitude to targets, Fz: *F*_(1,354)_ = 4.354, *p* = 0.038; Cz: *F*_(1,364)_ = 4.465, *p* = 0.035; Pz: *F*_(1,366)_ = 8.902, *p* = 0.003, as well as the N1 amplitude to standard tones at Pz: *F*_(1,370)_ = 5.997, *p* = 0.015, all of which were lower in the patients with MDD compared to the healthy controls. Overall, the healthy controls showed no age-related differences in the N1 amplitude, while in the MDD group, participants in the oldest subgroup elicited the smallest frontal N1 responses to standard tones compared to the other two subgroups (*p < 0.001)*.

### N1 latency

There were no significant Group, Age, or Sex main effects for the N1 latency. However, the main effect of Stimulus, *F*_(1,1089)_ = 8.809, *p* = 0.003, $\eta_{p}^{2}$ = 0.008, and the interactions of Stimulus × Electrode, *F*_(2,2198)_ = 7.625, *p* < 0.001, $\eta_{p}^{2}$ = 0.007, and Stimulus × Sex, *F*_(1,1089)_ = 4.176, *p* = 0.041, $\eta_{p}^{2}$ = 0.004, were significant. Follow-up tests noted that all participants elicited faster N1 responses for target than standard stimuli at Cz, *t*_(1,169)_ = 3.014, *p* = 0.003, *d* = 0.088; and Pz, *t*_(1,187)_ = 2.423, *p* = 0.016, *d* = 0.070. Stimulus differences in the timing of the N1 response did not reach significance at Fz.

Sex differences were present in the form of slightly faster N1 latencies in response to the standard tones in females vs. males, *F*_(1,1202)_ = 5.672, *p* = 0.017 (107.66 vs. 109.56 ms). No sex differences were observed in response to the target tones. Furthermore, only males demonstrated faster N1 latencies to targets than standards, *t*_(485)_ = 3.041, *p* = 0.002, *d* = 0.138 (107.74 vs. 109.56 ms).

### P300 amplitude

There were main effects of Group, *F*_(1,1089)_ = 7.529, *p* = 0.006, $\eta_{p}^{2}$ = 0.007; Sex, *F*_(1,1089)_ = 19.459, *p* < 0.001, $\eta_{p}^{2}$ = 0.018; and Stimulus, *F*_(1,1089)_ = 942.029, *p* < 0.001, $\eta_{p}^{2}$ = 0.464, as well as Stimulus × Group, *F*_(1,1089)_ = 9.139, *p* < 0.001, $\eta_{p}^{2}$ = 0.028; Stimulus × Sex, *F*_(1,1089)_ = 31.468, *p* < 0.001, $\eta_{p}^{2}$ = 0.028; Stimulus × Age Groups × Sex, *F*_(2,1089)_ = 3.284, *p* = 0.038, $\eta_{p}^{2}$ = 0.006; Stimulus × Electrode, *F*_(2,2178)_ = 779.650, *p* < 0.001, $\eta_{p}^{2}$ = 0.417; Stimulus × Electrode × Group, *F*_(2,2178)_ = 3.643, *p* = 0.026, $\eta_{p}^{2}$ = 0.003; Stimulus × Electrode × Sex, *F*_(2,2178)_ = 3.445, *p* = 0.032, $\eta_{p}^{2}$ = 0.003; Stimulus × Electrode × Age interactions; *F*_(4,2178)_ = 50.304, *p* = 0.003, $\eta_{p}^{2}$ = 0.085; and Stimulus × Electrode × Group × Age interactions, *F*_(4,2178)_ = 2.519, *p* = 0.039, $\eta_{p}^{2}$ = 0.005.

Further testing of the Stimulus × Electrode × Sex interaction revealed that both males and females elicited larger P300 amplitudes to targets than standards at each of the three analyzed locations (*p* < 0.001). Larger P300 amplitudes in response to targets were recorded in males vs. females at the Pz, *F*_(1,1199)_ = 29.614, *p* < 0.001; Cz, *F*_(1,1186)_ = 29.397, *p* < 0.001; and Fz, *F*_(1,1131)_ = 12.461, *p* < 0.001. Females also elicited smaller P300 amplitudes than males in response to standards at Cz, *F*_(1,1204)_ = 12.053, *p* < 0.001.

Analysis of the Stimulus × Electrode × Group × Age interaction identified no age-related differences in the P300 response of the healthy controls. In the MDD group, age differences were observed for the standard tone responses at the Fz site, *F*_(2,903)_ = 7.294, *p* < 0.001, where the oldest subgroup (46–65 years) elicited a larger response compared to the younger subgroups (vs. 18–30: *F*_(1,639)_ = 11.549, *p* < 0.001, vs. 31–45: *F*_(1,539)_ = 6.950, *p* = 0.009).

Clinical group differences were observed among 18–30-year-olds in response to targets at the Pz, *F*_(1,492)_ = 21.287, *p* < 0.001, with larger amplitudes in healthy controls than in the MDD group. A similar pattern was evident in the oldest subgroup, 46–65 years, *F*_(1,366)_ = 4.235, *p* = 0.040. No clinical group differences reached significance in the middle-age subgroup, 31–45 years. Further exploratory analyses revealed that brain responses to targets of patients in the youngest MDD subgroup were not statistically different from those of healthy controls age 31 years or older.

### P300 latency

There were main effects of Group, *F*_(1,1089)_ = 15.174, *p* < 0.001, $\eta_{p}^{2}$ = 0.014; Age, *F*_(2,1089)_ = 13.310, *p* < 0.001, $\eta_{p}^{2}$ = 0.024; and Stimulus, *F*_(1,1089)_ = 30.445, *p* < 0.001, $\eta_{p}^{2}$ = 0.027, as well as interactions of Stimulus × Age, *F*_(2,1089)_ = 36.538, *p* < 0.001, $\eta_{p}^{2}$ = 0.063; Stimulus × Sex, *F*_(1,1089)_ = 8.397, *p* = 0.004, $\eta_{p}^{2}$ = 0.008; Stimulus × Electrode, *F*_(2,2178)_ = 89.322, *p* < 0.001, $\eta_{p}^{2}$ = 0.076; Stimulus × Electrode × Age, *F*_(4,2178)_ = 8.774, *p* < 0.001, $\eta_{p}^{2}$ = 0.016; and Stimulus × Electrode × Group × Age, *F*_(4,2178)_ = 3.045, *p* = 0.016, $\eta_{p}^{2}$ = 0.006. Follow-up analyses noted sex differences in response to targets only, *F*_(1,1116)_ = 15.162, *p* < 0.001, with faster latencies in females than males (356.72 vs. 366.77 ms).

The MDD group produced P300 responses with longer latencies than the healthy controls in response to both standard and target stimuli. The interaction effect in the combined sample was due to faster P300 responses to target vs. standard stimuli at Cz, *t*_(1169)_ = 6.532, *p* < 0.001, *d* = 0.191; and Fz, *t*_(1120)_ = 14.310, *p* < 0.001, *d* = 0.427. No significant stimulus differences in the timing of the P300 response were observed at Pz (*p* = 0.385).

Age-related differences were observed in the healthy control group for the responses to targets at Pz, *F*_(2,307)_ = 4.382, *p* = 0.013, due to the longer latency in the oldest (46–65 years) subgroup compared to the younger ages, and for the standard stimuli at Fz, *F*_(2,309)_ = 30.049, *p* < 0.001, and Cz, *F*_(2,309)_ = 10.737, *p* < 0.001, due to the shorter latencies in the oldest subgroup. In the MDD group, age-related differences were present for all stimuli and electrode locations, with the increasing age resulting in progressive shortening of the latency to standards and an increase in latency to targets.

### Brain-behavior associations with clinical measures

The large sample size in the current study afforded high statistical power to detect multiple significant correlations among the ERP measures sensitive to the clinical group differences (N1 amplitude, P300 amplitude and latency) and the behavioral measures of depression symptoms. However, most of the correlations were exceedingly small, with values less than 0.1, raising questions about their practical significance. Therefore, we considered only the results with at least a small effect size (r = 0.15 or greater).

#### N1 response

Brain-behavior correlations for the N1 response at the Cz location are presented in Table 2. The N1 is commonly observed to be maximal at the Cz and is thought to index the automatic processing of stimulus physical characteristics and contribute to preattentive change detection (Key et al., 2005). Reduced N1 amplitudes to target tones at Cz in the 18–30-year-olds were associated with higher depressive symptoms as measured by the HDRS, SOFAS, QIDS, DASS Depression subscale, and BRISC Negativity Bias scale. In the 31–45-year-olds, reduced N1 amplitudes to targets at Cz were related to HDRS and SOFAS scores only. A different pattern was observed in the oldest subgroup, where reduced central N1 responses to standard tones were related to higher DASS scores on anxiety and stress subscales. In the combined sample, only the association between the smaller central N1 responses to targets and lower SOFAS scores reached the small effect size threshold.

**Table 2:** Correlations between the N1 amplitude at the Cz location in response to standard and target sounds and behavioral measures.

	**Standard**			**Target**		
**18–30 years**	**Pearson r**	**p**	**n**	**Pearson r**	**p**	**n**
Age	0.08	0.07	494	0.09	0.05	487
MDD duration	0.01	0.84	354	0.01	0.91	347
HDRS	0.04	0.40	494	**0.16**	**<0.001**	**487**
SOFAS	−0.07	0.14	494	**−0.20**	**<0.001**	**487**
QIDS	0.06	0.17	473	**0.19**	**<0.001**	**466**
DASS Anxiety	0.07	0.11	469	0.14	<0.001	462
DASS Depression	0.10	0.04	470	**0.19**	**<0.001**	**463**
DASS Stress	0.04	0.35	469	0.14	<0.001	462
BRISC Emotional Resilience	−0.04	0.38	472	−0.13	<0.001	465
BRISC Negativity Bias	−0.07	0.13	470	**−0.17**	**<0.001**	**463**
BRISC Social Skills	−0.10	0.02	476	−0.13	<0.001	469
**31–45 years**						
Age	0.02	0.77	342	0.01	0.90	335
MDD duration	0.07	0.28	257	0.04	0.56	252
HDRS	0.04	0.49	342	**0.16**	**<0.001**	**335**
SOFAS	−0.05	0.38	342	**−0.17**	**<0.001**	**335**
QIDS	0.00	0.94	321	0.09	0.10	316
DASS Anxiety	−0.03	0.63	316	0.05	0.42	310
DASS Depression	0.01	0.89	318	0.03	0.58	312
DASS Stress	−0.04	0.48	318	0.04	0.48	311
BRISC Emotional Resilience	0.04	0.45	324	−0.10	0.08	318
BRISC Negativity Bias	−0.01	0.81	320	−0.05	0.39	314
BRISC Social Skills	0.02	0.69	324	−0.08	0.16	318
**46–65 years**						
Age	0.14	0.01	369	0.06	0.28	365
MDD duration	0.05	0.40	267	0.09	0.13	263
HDRS	0.09	0.09	369	0.11	0.03	365
SOFAS	−0.10	0.06	369	−0.08	0.12	365
QIDS	0.10	0.06	346	0.11	0.04	341
DASS Anxiety	**0.16**	**<0.001**	**344**	0.08	0.15	341
DASS Depression	0.06	0.25	344	0.04	0.46	341
DASS Stress	**0.15**	**0.01**	**344**	0.11	0.05	341
BRISC Emotional Resilience	−0.08	0.14	348	−0.10	0.05	345
BRISC Negativity Bias	−0.12	0.03	346	−0.09	0.09	343
BRISC Social Skills	−0.04	0.42	348	0.00	0.94	345
**Combined**						
Age	0.07	0.02	1,205	0.04	0.22	1,187
MDD duration	0.06	0.07	878	0.04	0.21	862
HDRS	0.05	0.06	1,205	0.14	<0.001	1,187
SOFAS	−0.07	0.01	1,205	**−0.15**	**<0.001**	**1,187**
QIDS	0.06	0.06	1,140	0.14	<0.001	1,123
DASS Anxiety	0.07	0.02	1,129	0.10	<0.001	1,113
DASS Depression	0.06	0.04	1,132	0.10	<0.001	1,116
DASS Stress	0.05	0.10	1,131	0.10	<0.001	1,114
BRISC Emotional Resilience	−0.02	0.45	1,144	−0.11	<0.001	1,128
BRISC Negativity Bias	−0.07	0.03	1,136	−0.11	<0.001	1,120
BRISC Social Skills	−0.05	0.10	1,148	−0.08	0.01	1,132

Bold font identifies correlations that meet the minimum criterion of a small effect size. See Supplementary Table 1 for correlation results at the Fz and Pz locations. MDD, major depressive disorder; HDRS, Hamilton Depression Rating Scale; SOFAS, Social and Occupational Functioning Assessment Scale; QIDS, Quick Inventory of Depressive Symptomatology; DASS, Depression Anxiety and Stress Scales; BRISC, Brain Resource Inventory of Social Cognition.

#### P300 response

For the P300 responses indexing involuntary (frontal) and goal-directed (centro-parietal) attention, brain-behavior correlations are presented in Table 3 (amplitude) and Table 4 (latency). The larger parietal P300 amplitudes to target tones were related to lower depression symptoms (HDRS, QIDS, SOFAS, DASS Depression, BRISC Negativity Bias) and lower DASS anxiety and stress levels in the youngest subgroup. No consistent associations were observed with the P300 latency measures for that age range.

**Table 3:** Correlations between the P300 amplitude at the Fz, Cz, and Pz locations in response to standard and target sounds and behavioral measures.

	**Standard**									**Target**								
	**Fz**			**Cz**			**Pz**			**Fz**			**Cz**			**Pz**		
**18–30 years**	**Pearson r**	**p**	**n**	**Pearson r**	**p**	**n**	**Pearson r**	**p**	**n**	**Pearson r**	**p**	**n**	**Pearson r**	**p**	**n**	**Pearson r**	**p**	**n**
Age	0.04	0.37	498	0.05	0.25	494	−0.01	0.89	498	0.09	0.05	462	−0.06	0.20	487	**−0.15**	**<0.001**	**493**
MDD duration	−0.01	0.81	358	0.01	0.79	354	−0.01	0.93	358	0.13	0.02	322	0.12	0.02	347	0.07	0.19	353
HDRS	0.09	0.05	498	0.03	0.52	494	−0.04	0.33	498	−0.05	0.30	462	−0.09	0.04	487	**−0.22**	**<0.001**	**493**
SOFAS	−0.09	0.05	498	−0.03	0.57	494	0.07	0.14	498	0.04	0.35	462	0.06	0.19	487	**0.20**	**<0.001**	**493**
QIDS	0.07	0.15	477	0.01	0.90	473	−0.11	0.02	477	−0.12	0.01	441	−0.14	<0.001	466	**−0.25**	**<0.001**	**472**
DASS Anxiety	0.03	0.58	473	0.04	0.44	469	−0.02	0.61	473	−0.11	0.02	437	**−0.15**	**<0.001**	**462**	**−0.22**	**<0.001**	**468**
DASS Depression	0.08	0.10	474	0.04	0.35	470	−0.08	0.07	474	−0.06	0.22	438	−0.09	0.04	463	**−0.21**	**<0.001**	**469**
DASS Stress	0.07	0.11	473	0.03	0.50	469	−0.05	0.33	473	−0.06	0.20	437	−0.09	0.06	462	**−0.18**	**<0.001**	**468**
BRISC Emotional Resilience	−0.10	0.03	476	−0.04	0.37	472	0.06	0.17	476	0.04	0.38	440	0.09	0.07	465	0.13	0.01	471
BRISC Negativity Bias	−0.08	0.09	474	−0.04	0.44	470	0.07	0.11	474	0.08	0.12	438	0.10	0.03	463	**0.20**	**<0.001**	**469**
BRISC Social Skills	−0.07	0.13	480	−0.03	0.52	476	0.02	0.66	480	0.07	0.14	444	0.08	0.09	469	0.13	<0.001	475
**31–45 years**																		
Age	0.02	0.73	344	0.09	0.11	342	0.04	0.47	344	0.08	0.15	315	−0.04	0.44	335	−0.06	0.30	340
MDD duration	0.10	0.13	259	0.06	0.31	257	0.10	0.10	259	0.00	0.98	232	0.03	0.62	252	0.06	0.37	257
HDRS	0.08	0.12	344	0.03	0.62	342	−0.06	0.29	344	−0.01	0.82	315	−0.01	0.83	335	−0.09	0.09	340
SOFAS	−0.08	0.15	344	0.00	0.96	342	0.08	0.13	344	0.06	0.27	315	0.05	0.38	335	0.13	0.01	340
QIDS	0.07	0.19	323	0.01	0.88	321	−0.06	0.31	323	0.01	0.93	296	−0.01	0.82	316	−0.09	0.13	321
DASS Anxiety	−0.02	0.69	318	−0.04	0.51	316	−0.05	0.38	318	−0.02	0.70	290	−0.04	0.52	310	−0.07	0.22	315
DASS Depression	0.03	0.59	320	0.02	0.68	318	−0.06	0.27	320	−0.01	0.94	292	0.02	0.71	312	−0.04	0.50	317
DASS Stress	0.02	0.79	320	−0.02	0.67	318	0.00	0.94	320	0.03	0.58	291	0.04	0.48	311	0.01	0.8	316
BRISC Emotional Resilience	−0.02	0.77	326	0.01	0.83	324	0.10	0.08	326	0.07	0.21	298	0.05	0.41	318	0.12	0.04	323
BRISC Negativity Bias	−0.05	0.37	322	−0.01	0.91	320	0.02	0.69	322	−0.01	0.93	294	−0.02	0.73	314	0.03	0.64	319
BRISC Social Skills	−0.05	0.35	326	0.03	0.63	324	0.05	0.35	326	0.14	0.01	298	0.12	0.03	318	0.12	0.04	323
**46–65 years**																		
Age	**0.16**	**<0.001**	**372**	0.13	0.01	369	0.06	0.24	371	0.06	0.24	355	0.02	0.73	365	**0.15**	**0.01**	**367**
MDD duration	0.06	0.33	270	0.03	0.67	267	0.07	0.23	269	0.03	0.60	253	0.03	0.58	263	0.09	0.15	265
HDRS	0.02	0.73	372	−0.04	0.45	369	−0.01	0.93	371	−0.12	0.03	355	−0.08	0.11	365	−0.12	0.03	367
SOFAS	−0.07	0.18	372	0.00	0.95	369	−0.03	0.57	371	0.08	0.12	355	0.06	0.26	365	0.10	0.06	367
**45–65 years**	**Pearson r**	**p**	**n**	**Pearson r**	**p**	**n**	**Pearson r**	**p**	**n**	**Pearson r**	**p**	**n**	**Pearson r**	**p**	**n**	**Pearson r**	**p**	**n**
QIDS	0.02	0.72	349	−0.03	0.62	346	0.03	0.54	348	−0.09	0.10	331	−0.08	0.15	341	−0.11	0.04	343
DASS Anxiety	−0.05	0.40	346	−0.06	0.28	344	−0.06	0.26	345	**−0.17**	**<0.001**	**329**	−0.13	0.02	341	**−0.16**	**<0.001**	**341**
DASS Depression	−0.03	0.63	347	−0.04	0.41	344	0.01	0.85	346	−0.05	0.33	330	−0.05	0.39	341	−0.08	0.16	342
DASS Stress	0.01	0.83	347	−0.02	0.72	344	−0.03	0.65	346	−0.06	0.28	330	−0.04	0.52	341	−0.06	0.27	342
BRISC Emotional Resilience	−0.06	0.24	351	0.00	0.95	348	−0.03	0.56	350	0.00	0.98	334	0.00	0.99	345	0.10	0.08	346
BRISC Negativity Bias	0.04	0.45	349	0.05	0.36	346	0.02	0.75	348	0.08	0.14	332	0.06	0.28	343	0.10	0.07	344
BRISC Social Skills	−0.01	0.85	351	0.03	0.64	348	0.03	0.58	350	−0.02	0.71	334	0.03	0.59	345	0.05	0.32	346
**Combined**																		
Age	0.14	<0.001	1,214	0.14	<0.001	1,205	0.04	0.21	1,213	**0.21**	**<0.001**	**1,132**	−0.05	0.1	1,187	−0.12	<0.001	1,200
MDD duration	0.10	<0.001	887	0.08	0.02	878	0.07	0.04	886	0.13	<0.001	807	0.02	0.54	862	0.01	0.77	875
HDRS	0.07	0.02	1,214	0.01	0.75	1,205	−0.03	0.25	1,213	−0.05	0.09	1,132	−0.07	0.02	1,187	**−0.16**	**<0.001**	**1,200**
SOFAS	−0.08	<0.001	1,214	−0.01	0.65	1,205	0.04	0.21	1,213	0.05	0.11	1,132	0.06	0.04	1,187	**0.16**	**<0.001**	**1,200**
QIDS	0.05	0.08	1,149	−0.01	0.87	1,140	−0.05	0.1	1,148	−0.07	0.02	1,068	−0.09	0	1,123	**−0.17**	**<0.001**	**1,136**
DASS Anxiety	−0.01	0.66	1,137	−0.02	0.61	1,129	−0.04	0.18	1,136	−0.11	<0.001	1,056	−0.11	<0.001	1,113	−0.14	<0.001	1,124
DASS Depression	0.03	0.29	1,141	0.01	0.75	1,132	−0.05	0.12	1,140	−0.04	0.21	1,060	−0.05	0.11	1,116	−0.12	<0.001	1,128
DASS Stress	0.03	0.25	1,140	0.00	0.91	1,131	−0.03	0.31	1,139	−0.04	0.18	1,058	−0.04	0.25	1,114	−0.09	<0.001	1,126
BRISC Emotional Resilience	−0.05	0.13	1,153	0.00	0.92	1,144	0.04	0.13	1,152	0.06	0.05	1,072	0.05	0.13	1,128	0.11	<0.001	1,140
BRISC Negativity Bias	−0.03	0.36	1,145	0.01	0.88	1,136	0.04	0.13	1,144	0.06	0.04	1,064	0.05	0.08	1,120	0.12	<0.001	1,132
BRISC Social Skills	−0.05	0.07	1,157	0.00	0.94	1,148	0.03	0.27	1,156	0.04	0.16	1,076	0.09	0	1,132	0.13	<0.001	1,144

Bold font identifies correlations that meet the minimum criterion of a small effect size. MDD, major depressive disorder; HDRS, Hamilton Depression Rating Scale; SOFAS, Social and Occupational Functioning Assessment Scale; QIDS, Quick Inventory of Depressive Symptomatology; DASS, Depression Anxiety and Stress Scales; BRISC, Brain Resource Inventory of Social Cognition.

**Table 4:** Correlations between the P300 latency at the Fz, Cz, and Pz locations in response to standard and target sounds and behavioral measures.

	**Standard**									**Target**								
	**Fz**			**Cz**			**Pz**			**Fz**			**Cz**			**Pz**		
**18–30 years**	**Pearson r**	**p**	**n**	**Pearson r**	**p**	**n**	**Pearson r**	**p**	**n**	**Pearson r**	**p**	**n**	**Pearson r**	**p**	**n**	**Pearson r**	**p**	**n**
Age	−0.08	0.08	498	0.00	0.97	494	0.00	0.96	498	0.01	0.84	462	0.04	0.39	487	0.03	0.54	493
MDD duration	**−0.18**	**<0.001**	**358**	−0.10	0.07	354	−0.07	0.20	358	0.02	0.70	322	0.00	1.00	347	−0.03	0.65	353
HDRS	0.07	0.11	498	0.07	0.12	494	0.11	0.02	498	0.06	0.21	462	0.09	0.06	487	0.05	0.31	493
SOFAS	−0.04	0.38	498	−0.05	0.26	494	−0.07	0.12	498	−0.06	0.23	462	−0.07	0.13	487	−0.04	0.40	493
QIDS	0.06	0.17	477	0.07	0.11	473	0.10	0.03	477	0.09	0.06	441	0.12	0.01	466	0.07	0.12	472
DASS Anxiety	0.04	0.40	473	0.10	0.04	469	0.13	0.00	473	0.07	0.17	437	0.10	0.03	462	0.08	0.08	468
DASS Depression	0.05	0.30	474	0.06	0.21	470	0.10	0.03	474	0.04	0.39	438	0.04	0.41	463	0.00	0.96	469
DASS Stress	0.02	0.63	473	0.08	0.10	469	0.12	0.01	473	0.06	0.25	437	0.04	0.38	462	0.00	0.96	468
BRISC Emotional Resilience	−0.05	0.33	476	−0.05	0.33	472	−0.11	0.01	476	−0.03	0.54	440	−0.04	0.46	465	0.02	0.72	471
BRISC Negativity Bias	−0.05	0.33	474	−0.08	0.10	470	−0.11	0.01	474	−0.04	0.43	438	−0.05	0.29	463	−0.01	0.87	469
BRISC Social Skills	−0.06	0.18	480	−0.07	0.12	476	−0.10	0.03	480	0.03	0.48	444	0.02	0.61	469	0.01	0.76	475
**31–45 years**																		
Age	−0.02	0.68	344	−0.01	0.79	342	0.00	0.95	344	0.02	0.67	315	−0.01	0.85	335	0.05	0.40	340
MDD duration	0.07	0.28	259	−0.06	0.33	257	0.05	0.39	259	−0.04	0.56	232	0.07	0.31	252	−0.02	0.82	257
HDRS	0.05	0.36	344	0.06	0.31	342	0.04	0.41	344	−0.05	0.42	315	0.01	0.90	335	0.05	0.34	340
SOFAS	−0.08	0.13	344	−0.06	0.29	342	−0.04	0.45	344	0.02	0.67	315	0.00	0.99	335	−0.06	0.26	340
QIDS	0.13	0.02	323	0.11	0.06	321	0.10	0.08	323	−0.05	0.43	296	−0.02	0.67	316	0.06	0.33	321
DASS Anxiety	0.08	0.17	318	0.14	0.01	316	0.12	0.04	318	−0.08	0.16	290	0.01	0.90	310	0.01	0.87	315
DASS Depression	0.12	0.03	320	0.13	0.02	318	0.11	0.06	320	−0.04	0.46	292	0.02	0.78	312	0.04	0.51	317
DASS Stress	**0.15**	**0.01**	**320**	**0.15**	**0.01**	**318**	0.11	0.04	320	−0.02	0.69	291	0.04	0.49	311	0.01	0.85	316
BRISC Emotional Resilience	−0.06	0.32	326	−0.06	0.26	324	0.00	0.99	326	0.04	0.52	298	0.02	0.69	318	0.00	0.96	323
BRISC Negativity Bias	−0.13	0.03	322	−0.13	0.02	320	−0.11	0.04	322	0.03	0.66	294	−0.03	0.64	314	−0.04	0.49	319
BRISC Social Skills	−0.11	0.05	326	−0.11	0.04	324	−0.10	0.06	326	0.02	0.68	298	0.02	0.67	318	0.01	0.83	323
**46–65 years**																		
Age	−0.14	0.01	372	−0.07	0.17	369	−0.06	0.29	371	0.13	0.02	355	**0.18**	**<0.001**	**365**	**0.29**	**<0.001**	**367**
MDD duration	−0.05	0.39	270	−0.05	0.40	267	0.05	0.44	269	−0.04	0.53	253	−0.06	0.34	263	−0.05	0.46	265
HDRS	**0.15**	**0.00**	**372**	0.08	0.11	369	0.06	0.25	371	0.02	0.70	355	0.09	0.08	365	0.11	0.03	367
SOFAS	−0.13	0.01	372	−0.05	0.33	369	−0.04	0.45	371	0.03	0.56	355	−0.04	0.45	365	−0.08	0.11	367
**46–65 years**	**Pearson r**	**p**	**n**	**Pearson r**	**p**	**n**	**Pearson r**	**p**	**n**	**Pearson r**	**p**	**n**	**Pearson r**	**p**	**n**	**Pearson r**	**p**	**n**
QIDS	**0.17**	**<0.001**	**349**	0.06	0.28	346	0.07	0.17	348	−0.03	0.57	331	0.04	0.46	341	0.09	0.09	343
DASS Anxiety	**0.15**	**0.01**	**346**	0.13	0.01	344	0.14	0.01	345	−0.02	0.66	329	0.03	0.58	341	0.10	0.06	341
DASS Depression	**0.15**	**0.01**	**347**	0.06	0.25	344	0.07	0.18	346	−0.03	0.66	330	0.03	0.56	341	0.06	0.29	342
DASS Stress	0.11	0.04	347	0.06	0.28	344	0.03	0.55	346	−0.08	0.17	330	−0.02	0.72	341	0.04	0.50	342
BRISC Emotional Resilience	−0.06	0.25	351	−0.04	0.44	348	−0.08	0.15	350	0.00	0.94	334	−0.03	0.62	345	−0.08	0.12	346
BRISC Negativity Bias	**−0.15**	**<0.001**	**349**	−0.08	0.12	346	−0.09	0.08	348	0.04	0.46	332	−0.02	0.73	343	−0.08	0.17	344
BRISC Social Skills	**−0.15**	**<0.001**	**351**	**−0.22**	**<0.001**	**348**	−0.12	0.03	350	0.07	0.23	334	0.01	0.83	345	−0.01	0.93	346
**Combined**																		
Age	**−0.34**	**<0.001**	**1,214**	**−0.24**	**<0.001**	**1,205**	−0.10	<0.001	1,213	0.07	0.02	1,132	0.10	<0.001	1,187	**0.24**	**<0.001**	**1,200**
MDD duration	**−0.18**	**<0.001**	**887**	**−0.17**	**<0.001**	**878**	−0.04	0.23	886	0.00	0.97	807	0.03	0.38	862	0.08	0.02	875
HDRS	0.08	0.01	1,214	0.06	0.03	1,205	0.07	0.01	1,213	0.02	0.45	1,132	0.07	0.02	1,187	0.07	0.01	1,200
SOFAS	−0.06	0.04	1,214	−0.04	0.17	1,205	−0.05	0.10	1,213	−0.01	0.79	1,132	−0.04	0.14	1,187	−0.07	0.02	1,200
QIDS	0.11	<0.001	1,149	0.08	0.01	1,140	0.09	0.00	1,148	0.02	0.56	1,068	0.06	0.06	1,123	0.07	0.02	1,136
DASS Anxiety	0.10	<0.001	1,137	0.13	<0.001	1,129	0.14	<0.001	1,136	0.01	0.64	1,056	0.06	0.05	1,113	0.05	0.07	1,124
DASS Depression	0.10	<0.001	1,141	0.08	0.01	1,132	0.09	<0.001	1,140	0.00	0.99	1,060	0.03	0.34	1,116	0.03	0.36	1,128
DASS Stress	0.09	<0.001	1,140	0.10	<0.001	1,131	0.10	<0.001	1,139	0.00	0.91	1,058	0.02	0.52	1,114	0.00	0.95	1,126
BRISC Emotional Resilience	−0.09	<0.001	1,153	−0.08	0.01	1,144	−0.08	0.01	1,152	0.01	0.70	1,072	0.00	0.93	1,128	0.01	0.66	1,140
BRISC Negativity Bias	−0.12	<0.001	1,145	−0.11	<0.001	1,136	−0.11	<0.001	1,144	0.00	0.91	1,064	−0.03	0.33	1,120	−0.02	0.47	1,132
BRISC Social Skills	−0.06	0.03	1,157	−0.10	<0.001	1,148	−0.09	<0.001	1,156	0.04	0.15	1,076	0.02	0.52	1,132	−0.01	0.77	1,144

Bold font identified correlations that meet the minimum criterion of a small effect size. MDD, major depressive disorder; HDRS, Hamilton Depression Rating Scale; SOFAS, Social and Occupational Functioning Assessment Scale; QIDS, Quick Inventory of Depressive Symptomatology; DASS, Depression Anxiety and Stress Scales; BRISC, Brain Resource Inventory of Social Cognition.

In the middle-age subgroup, no correlations with the P300 amplitude reached the small effect threshold, while longer frontal and central P300 latency to standard tones were associated with higher DASS stress scores.

In the oldest subgroup, larger frontal and parietal P300 amplitude to targets were related to lower DASS anxiety scores. Furthermore, consistent with the pattern observed for the N1 amplitude, responses to standard tones were more informative about the depressive symptoms in the oldest subgroup: longer latency of the frontal P300 was related to higher scores on HDRS, QIDS, DASS Depression and DASS Anxiety, as well as lower scores on BRISC Negativity Bias and Social Skills.

In the combined sample, only the associations between the higher parietal P300 amplitude to targets and lower depressive symptoms (HDRS, SOFAS, QIDS) reached the small effect size threshold.

## Discussion

The primary goal of this reanalysis of an existing large data set was to systematically examine whether combinations of neural biomarkers, demographic characteristics, and clinical features could be used to define subtypes of MDD patients to inform sample selection and stratification strategies for MDD drug development. A key objective was to determine whether the N1 and P300 components of ERP responses are sensitive to neural pathophysiology in MDD and whether they may serve as informative neural biomarkers of MDD. The findings from the classic auditory oddball task indicated that all participants, regardless of age, clinical group membership, or depression symptom severity, could discriminate between the two tones varying in frequency as reflected by the larger N1 and P300 amplitudes in response to the target compared to the standard stimuli. However, compared to the healthy controls, the patients with MDD generated smaller N1 and P300 response amplitudes and longer P300 latencies for both stimulus conditions, suggesting alterations in sensory and goal-directed attentional processes. Age was a significant modulator of these neural differences between patients with MDD and healthy controls.

Analysis of the N1 responses reflecting early sensory auditory processing (encoding of acoustic features and preattentive change detection: Martin et al., 1999; Näätänen et al., 2011) noted smaller than typical N1 amplitudes to targets in patients with MDD at all three analyzed scalp locations. The magnitude of stimulus discrimination (target-standard amplitude difference) was also reduced in the MDD group compared to the healthy controls, suggesting lower than typical sensitivity to acoustic frequency differences and/or reduced top-down allocation of neural processing resources to the sensory stimuli. Larger N1 amplitudes have been previously reported in studies requiring active attention to the stimuli compared to passive exposure (Hillyard et al., 1973; Näätänen et al., 1978; Coch et al., 2005). Conversely, no significant clinical group differences were observed for the speed of early auditory processing, as indexed by the N1 latency.

Brain-behavior correlations allowed us to investigate the functional significance of the N1 amplitude reduction in the MDD group. The results revealed consistent associations between the reduction in the central N1 amplitudes to target tones and the increased severity of depression symptoms, as measured by clinician observations and by self-report in the 18–30-year-old subgroup. In the 31–45-year-olds, such associations were observed only with clinician ratings. Of note, in the oldest participants, the central N1 response to targets did not relate to any clinical symptoms of MDD. Instead, reduced N1 amplitudes to standard stimuli at central sites, indicating more general tuning out of sensory inputs, were related to increased anxiety and stress, as measured by the DASS. Previously, increased state and trait anxiety had been associated with reduced sensory registration in non-MDD populations (Engel-Yeger and Dunn, 2011). Similarly, increased auditory sensory difficulties have been related to greater depression and anxiety symptoms in older adults (Simning et al., 2019).

In combination, these results replicate prior findings of N1 amplitude reductions in MDD (e.g., Burkhart and Thomas, 1993) and suggest that patients of all ages with increased depression symptoms may exhibit reduced bottom-up sensory processing and/or less effective top-down attention allocation needed to detect unexpected changes in the auditory environment. In addition, age appears to be an important contributor to the MDD phenotype and should be considered in the context of treatment planning.

Analysis of the P300 responses, indexing goal-directed (central, parietal) and involuntary (frontal) attention allocation to the auditory target detection task revealed the expected larger amplitudes to the infrequent targets compared to the standard tones in all participants. Replicating prior findings of the reduced P300 amplitudes in patients with MDD (see Mumtaz et al., 2015 for review), the MDD group elicited smaller than typical responses to targets, reflecting reduced attention allocation to task performance. No group differences were observed for the standard stimuli.

Furthermore, clinical group differences were most clearly observed for the youngest (18–30 years) and oldest (46–65 years) subgroups. Brain-behavior associations noted that reduced parietal P300 amplitudes in response to target tones in the youngest age subgroup were related to increased symptoms of depression (both clinician-observed and self-reported) as well as to higher anxiety and stress scores, consistent with prior reports of high comorbidity of anxiety and depression (e.g., Lamers et al., 2011). In the oldest age subgroup, a similar association was observed between the self-reported anxiety and P300 amplitudes to targets at parietal and frontal locations. The reduction in the P300 amplitude was recently reported to be predictive of future depression severity in patients with MDD (Santopetro et al., 2021). Previously, smaller P300 responses were observed in patients with depression and anxiety compared to patients with anxiety alone (Bruder et al., 2002; Enoch et al., 2008); thus, the current results are likely driven by the depression features.

In addition, exploratory follow-up analyses noted that the parietal P300 response amplitudes to targets in the youngest MDD patients (18–30-year-olds) were not significantly different from those in the older healthy controls. These results extend previous reports of associations between accelerated cognitive decline in aging and depression symptoms (Panza et al., 2009; Oi, 2017; Sacchet et al., 2017), and similar to the N1 findings, highlight the importance of considering the participants’ age.

Analyses of the P300 response latencies noted the expected faster responses to targets than standard tones in all participants, as well as prolonged latencies in the MDD group compared to the healthy controls for both stimulus types. The latter is consistent with the generalized slowing in information processing and reduced sustained attention performance (e.g., Nebes et al., 2000; van der Meere et al., 2007), which have been reported to explain some of the key symptoms in MDD (Piani et al., 2022). In the healthy controls, age-related differences were observed only for the target P300 latency with longer values in the oldest compared to the younger two subgroups. In the MDD group, the latency for the rare targets progressively increased across the three age subgroups while the latency for frequent standard stimuli decreased with increasing age.

A novel contribution of this study is the identification of the anterior P300 response latency, which indexes the speed of involuntary attentional responses, as a candidate biomarker sensitive to MDD symptom severity in middle- and older-age adults. Brain-behavior associations indicated that the timing of the frontal P300 response to standard stimuli was related to the severity of depression symptoms only in the oldest subgroup (46–65 years), suggesting that increased depression was associated with less efficient processing of the frequent stimuli. It also correlated with stronger negativity bias and lower social skills. In the 31–45-year-olds, prolonged frontal and central P300 latencies to standard tones were related to increased scores on the stress subscale, while no systematic relationships with the behavioral measures were observed for the youngest subgroup. The anterior scalp distribution of the P300 latencies involved in these brain-behavior associations suggests that MDD symptoms may interfere with the speed of involuntary attention allocation to regularly presented stimuli, possibly indicating slower memory trace formation for the standard tones, resulting in less efficient stimulus categorization. These observations are consistent with prior reports of a compensatory frontal shift in the P300 topography in older adults (van Dinteren et al., 2018) and serve as additional evidence of potentially accelerated cognitive aging in MDD (see also Oi, 2017; Sacchet et al., 2017; Lorenzo et al., 2022).

Similar auditory ERP alterations, especially for the P300 response, have been previously observed in other clinical groups, such as schizophrenia and dementia (e.g., Hedges et al., 2016; Hamilton et al., 2019). Of note, those populations also have high rates (up to 50%) of comorbid depression diagnoses or preclinical depression symptoms that are often present early in the disease progression (e.g., Häfner et al., 2005; Cipriani et al., 2015). In the current study, the participants with MDD as well as the healthy controls were free of schizophrenia, bipolar, obsessive-compulsive, or posttraumatic stress disorders, and substance abuse (see Williams et al., 2011). Furthermore, the two groups were matched on age, sex, and education. Therefore, we attribute the observed group differences in the auditory ERPs to the presence of MDD.

Our results extended the original report by van Dinteren et al. (2015) that identified different alterations of neural responses in the chronologically younger vs. older subgroups of patients with MDD compared to the healthy controls. These findings are also consistent with the observation that symptoms of depression vary with age (Brown et al., 2016). Time since birth is the most readily available and therefore commonly used objective measure of age. However, prior studies (e.g., Han et al., 2018), as well as our results, suggest that patients with MDD may be biologically older than their chronological age, and the latest reports note a significant overlap between the biological alterations in MDD and those commonly observed in aging (Lorenzo et al., 2022). The lack of an objectively quantified biological age is a limitation of the current analysis. Thus, future studies should consider including an estimate of biological age based on the clinical tests of multiple physiological systems (e.g., immune, metabolic, cardiovascular, renal; Belsky et al., 2015; Jazwinski and Kim, 2019). Although correlated, biological and chronological age measures would contribute distinct information. Indeed, in older adults, biological age had been a stronger predictor of depressive symptoms than chronological age (Brown et al., 2018). Identifying the unique contributions of biological and chronological age could facilitate treatment planning.

Despite observing several significant differences in the auditory ERP responses between the healthy controls and participants with MDD, as well as the numerous brain-behavior associations, it is important to note that the observed effect sizes are small. In other words, although our large, well-characterized sample provided sufficient statistical power to detect these differences, similar findings may not be observable in smaller samples. Furthermore, the small effect sizes suggest that the reported clinical group differences in neural activity recorded during the performance of an active auditory oddball task, while informative, may not be sufficient on their own to fully characterize individual differences related to MDD. Considering additional subject characteristics such as age (e.g., chronological, biological, etc.) can increase the utility of neural data.

In conclusion, the large iSPOT-D sample allowed us sufficient sensitivity to conclude that auditory N1 and P300 responses in an oddball task reflect patterns of neural functioning that differ between individuals with MDD and healthy controls. In addition, age emerged as a critical contributor to the observed results, as reflected by a shift from clinical group differences specific to target- or change-related processing (observed in all age groups) to more broad differences involving both target and standard stimuli (observed mainly in the older participants). The patterns of brain-behavior correlations confirmed functional associations between the depression symptoms and the N1 and P300 response characteristics, as well as noted the increasing contributions of anxiety and stress measures in the older subgroups. Future studies may explore the role of DASS Anxiety and Stress scale scores as mediators between the altered sensory and attentional neural processes and MDD severity (Wigham et al., 2015; Engel-Yeger et al., 2016). Overall, these findings support the use of auditory N1 and P300 responses along with age as an effective means of stratifying the clinical population with MDD, with the goal of reducing heterogeneity and therefore facilitating a more efficient design of future treatment studies.

## Data Availability Statement

The data analyzed in this study is subject to the following licenses/restrictions: the data analyzed in this study were obtained from Total Brain Ltd. Requests to access these datasets should be directed to Evian Gordon, evian.gordon@totalbrain.com.

## Author Contributions

AK designed the analysis approach, performed statistical tests and functional interpretation of the results, and contributed to writing this report. TT-W and DS conceived the study and contributed to interpretation of the results and writing this report. All authors contributed to the article and approved the submitted version.
